# Death of Dr. J. F. Y. Paine

**Published:** 1912-12

**Authors:** Chas. W. Trueheart, H. P. Cooke, Geo. H. Lee


### Death of Dr. J. F. Y. Paine.

Dr. John Fannin Young Paine, who died on October 2nd of this year at his home in Charlestown, W. Va., where he had removed after his retirement from active practice about three years ago, was for nearly forty years in the practice of medicine in Galveston and a useful and influential member of the Galveston County Medical Society and its predecessor, the Galveston Medical Club.

Therefore, the members of this society, in meeting assembled, on October 25, 1912, as a slight, but much merited, tribute to his memory, hereby resolve that the following facts connected with his useful and eventful life and thoughts concerning his character and life-work be spread upon the minutes of this meeting, and that copies of this resolution be sent to his bereaved family.

Dr. Paine was born in Louisiana, August 16, 1840; was educated in the schools of that State and of Mississippi, graduating from the Centenary College in the latter State. His medical education was begun at the University of Pennsylvania, but was completed at the University of Louisiana (now Tulane University), where he graduated in 1861.

He served in the Confederate army as field and staff surgeon, and was present at Mobile when Fort Morgan was bombarded by the Federal fleet, and was wounded in the foot by a piece of exploded shell in the action.

After the war Dr. Paine located for a time in Mobile, where he married Miss Bettie Estes. Later he removed to Texas and resided for several years at Ennis. In 1876 he came to Galveston to accept the chair of obstetrics and gynecology in the Texas Medical College, the first institution for medical education in this State. He afterward was the dean of the faculty, until the school was discontinued, in 1881, when the election had just determined that Galveston would be the location of the medical department of the new State University, just then being organized, and it was thought the new medical department would be inaugurated at once.

In 1885 he was called to the chair of materia medica and therapeutics in the Medical Department of Tulane University of Louisiana, at New Orleans, which call he accepted. He filled that chair for one year, after which he returned to this city.

In 1888 he was active in the reorganization of the Texas Medical College, which movement was designed to influence the Board of Regents of the University to inaugurate the School of Medicine of the State University. Dr. Paine was especially active in procuring the liberal donations of funds with which to equip and operate the Texas Medical College. He filled a chair in the college and was for a time dean of the faculty, until that college gave way to the newly organized Medical Department of the University of Texas.

He was the first member chosen for the medical faculty of the new School of Medicine, having been elected to the chair of obstetrics and gynecology, which chair he filled with distinguished ability, until failing health compelled him to give up active teaching. The Board of Regents then recognized his meritorious service by electing him emeritus professor of that chair.

He was the first dean of the faculty of the School of Medicine.

His personal friendship and his influence were probably largely responsible for giving such direction to that liberal and munificent bequest which resulted in the building of the John Sealy Hospital. He served on the building committee of that hospital, and was continuously on the visiting staff until his removal from the city.

He filled other positions of honor and distinction, being for one year (1888 to 1889) president of the Texas State Medical Association.

Dr. Paine was a man of unusual intellectual ability, laborious and painstaking as a student, of splendid personal appearance, with manners and bearing always courteous and dignified, and with ideals that kept his life ever upon a high plane.

As teacher of medicine, his example and influence embodied in his personality, and lectures with his method of thoroughness, and his high ideals, served to impress his students and to stimulate in them their aspirations for the nobler conceptions of their profession.

As a practitioner of medicine, he was thorough and accurate in his diagnosis, unsurpassed in the breadth and fullness of his information, and tireless in his efforts to serve the sick, whether poor or rich.

He exemplified in his life and character not only a high order of citizen, friend and teacher, but also the splendid gentleman of the old school and the beloved family doctor, than whose life none could be more useful or more filled with love for his fellow men.

CHAS. W. TRUEHEART.
H. P. COOKE.
GEO. H. LEE.

The faculty of the Medical Department of the University of Texas also adopted a series of resolutions in testimony of the worth and merit of their deceased colleague.

